# Liposomal cytarabine in the prophylaxis and treatment of CNS lymphoma: toxicity analysis in a retrospective case series study conducted at Polish Lymphoma Research Group Centers

**DOI:** 10.1007/s12032-015-0520-3

**Published:** 2015-02-26

**Authors:** Wojciech Jurczak, Renata Kroll-Balcerzak, Sebastian Giebel, Maciej Machaczka, Agnieszka Giza, Tomasz Ogórka, Szymon Fornagiel, Justyna Rybka, Tomasz Wróbel, Beata Kumiega, Aleksander B. Skotnicki, Mieczysław Komarnicki

**Affiliations:** 1 Department of Haematology, Jagiellonian University, 36 Kopernika str., 30-501 Kraków, Poland; 2 Department of Haematology, Poznan University of Medical Sciences, 84 Szamarzewskiego str., 60-569 Poznan, Poland; 3 Gliwice Branch, Department of Bone Marrow Transplantation and Onco-Haematology, Maria Sklodowska-Curie Memorial Cancer Center and Institute of Oncology, Wybrzeże Armii Krajowej 15, 44-101 Gliwice, Poland; 4 Memorial Sloan Kettering Cancer Center, New York, NY USA; 5 Division of Haematology, Department of Medicine at Huddinge, Karolinska Institutet and Karolinska University Hospital, Hälsovägen, Flemingsberg, 141 86 Stockholm, Sweden; 6 Department of Haematology, Wrocław University of Medicine, Wyb. Pasteura 4, 50-367 Wrocław, Poland; 7 Departament of Haematooncology, Markiewicz Memorial Oncology Center Brzozow, 18 Ks. Bierkowskiego str., 36-200 Brzozów, Poland

**Keywords:** Intrathecal liposomal cytarabine, CNS lymphoma, Prophylaxis

## Abstract

Lymphomas with primary or secondary involvement of central nervous system (CNS) have poor prognosis despite specific treatment protocols which include whole brain radiotherapy and high-dose systemic and/or intrathecal chemotherapy. Toxicity of intrathecal liposomal cytarabine-based regimens collected between November 2006 and January 2012 was assessed retrospectively. Data from 120 adult lymphoma patients with, or at high risk of CNS involvement who received intrathecal liposomal cytarabine-based regimens at six Polish Lymphoma Research Group centres between November 2006 and January 2012 were assessed retrospectively. Patients were divided into three cohorts: A (high risk of CNS disease, *n* = 88), B (cerebrospinal fluid pleocytosis without neurological symptoms or pathological imaging findings, *n* = 7), and C (CNS disease/neurological involvement; *n* = 25). In all examined groups, toxicity of treatment was found to be acceptable (including the prophylactic setting). None of the patients in cohorts A or B who took intrathecal liposomal cytarabine 50 mg, repeated every 2–4 weeks (mean 3.8 doses) had experienced a CNS relapse at a median follow-up time of 3 years. Patients in cohort C had a 76 % overall neurological response rate (including a 40 % complete response rate) and median overall survival of 4.8 years. Regimens incorporating liposomal cytarabine seem to be safe and effective treatments for lymphomas with CNS involvement.

## Introduction

Central nervous system (CNS) involvement is a serious, life-shortening complication of non-Hodgkin’s lymphoma (NHL), with a prevalence ranging from <3 % in indolent to 30 % in aggressive forms of the disease [1–4]. Current treatment options for CNS lymphoma (high-dose CNS-penetrating chemotherapy and/or whole brain radiation therapy; WBRT) are rarely curative [3, 4]. Moreover, toxicity of treatment is commonly at unacceptable level. Primary CNS lymphoma (PCNSL) has a particularly poor prognosis, with approximately 90 % of relapsing/refractory cases [5]. Aggressive NHL subtypes like DLBCL with secondary CNS involvement have a median survival time of 2–6 months; <25 % of the patients are alive 1 year after diagnosis [2, 3, 5].

CNS-directed therapy is a widely accepted standard in the first line therapy of lymphoblastic and Burkitt’s lymphomas, where the risk of CNS relapses exceeds 20 % [6]. Most cases, however, occur in patients with diffuse large B cell lymphoma (DLBCL), peripheral T cell lymphoma and other “intermediate grade lymphomas” which have a cumulative incidence of secondary CNS involvement at a rate of 1–26 % at 5 years, depending on the risk group [1, 7–10].

CNS prophylaxis in high-risk asymptomatic cases might, therefore, be recommended as a standard practice after a risk assessment including the analysis of effectiveness and potential toxicity of prophylactic agents. R-CHOP (rituximab, cyclophosphamide, doxorubicin, vincristine, and prednisone), the most commonly used protocol in DLBCL has poor CNS penetration and has no effect on the CNS relapse rate [9, 11–15].

The marginally decreased incidence of CNS relapses in the R-CHOP era might be related to decreased number of systemic relapses [12, 16]. Although intrathecally administered anti-metabolites methotrexate (MTX) and/or cytarabine reduce relapse rates, their efficacy is compromised by pharmacokinetic issues. The relatively short half-lives of MTX and cytarabine (3.4 and 4.5 h, respectively) may result in inadequate biodistribution—homogenous, cytotoxic concentrations in the entire leptomeningeal compartment are difficult to achieve [17]. To achieve constant therapeutic concentrations of MTX and/or cytarabine, 2–3 lumbar punctures would have been necessary every week, which is uncomfortable and inconvenient for the patient. Less frequent intrathecal cytostatic administration, widely used in DLBCL patients is probably suboptimal, even in prophylaxis, as reflected by some study results [11, 12, 18].

Liposomal cytarabine (DepoCyte^®^) is a slow-release formulation that maintains cytotoxic concentrations in the cerebrospinal fluid (CSF) for at least 14 days in adults after a single intrathecal injection, achieving extensive distribution throughout the neuraxis. This formulation comprises aqueous chambers of the active drug (cytarabine) encapsulated within spherical, biodegradable lipid bi-layers which form 3- to 30-µm-diameter particles (DepoFoam), suspended in a 0.9 % sodium chloride solution. The CNS half-life of an intrathecal liposomal cytarabine in a 12.5–75 mg dose is 29- to 77-fold greater than a 30 mg dose of intrathecal free cytarabine (100–263 vs. 3.4 h) [19, 20]. Furthermore, cytotoxic concentrations (≥0.1 mg/ml) are maintained in the CSF for >14 days in most adults following a single liposomal cytarabine dose compared to <24 h for free cytarabine. Intrathecal liposomal cytarabine also demonstrated a significantly higher response rate and greater quality of life improvement than intrathecal cytarabine or MTX in the treatment of lymphomatous meningitis [21–23]. The treatment toxicity issue is still under investigation; this is particularly important for the prophylaxis setting where at least some of the patients may not have CNS involvement.

This retrospective analysis evaluated the tolerability of liposomal cytarabine-based regimens in the treatment or prevention of CNS involvement in patients with aggressive NHL (mainly high-risk DLBCL). It addresses the adverse events (AEs) of liposomal cytarabine including serious ones such as myelopathy. Other aspects will include an efficacy analysis of CNS lymphoma treatment and whether the benefits of prophylaxis outweigh the risks.

## Materials and methods

Toxicity and efficacy of intrathecal liposomal cytarabine (Depocyte)-based regimens, administered in 120 adult lymphoma patients with, or at high risk of CNS involvement who received liposomal cytarabine regimens at six Polish Lymphoma Research Group (PLRG) centers between November 2006 and January 2012 were analysed retrospectively.

Each patient underwent clinical assessments, neurological examinations, complete blood cell and biochemical profiling, a bone marrow biopsy, whole-body computed tomography (CT), and/or nuclear magnetic resonance (NMR) to confirm systemic disease status and/or the presence of CNS involvement. Complete blood cell count, biochemical profiling, CSF analysis (cell count and cytology), and a neurological examination were conducted prior to each liposomal cytarabine administration. The cytological method is less accurate than flow cytometry, so minor CSF involvement could have been missed. AEs, classified according to version 4.0 of the Common Terminology Criteria for Adverse Events of the National Cancer Institute (NCI-CTCAE v 4.0), were assessed and recorded at each hospital visit. Event-free survival (EFS) was assessed clinically and by CT/NMR imaging and defined as the time from the first intrathecal injection of liposomal cytarabine until death, disease progression or relapse. Overall survival (OS) was measured from the time of the first dose of liposomal cytarabine until death or the last observation. Descriptive statistics were used to summarize patient characteristics. Time-to-event curves and median time-to-event (EFS, OS) values were generated using the Kaplan–Meier method.

Depocyte was used for the prophylaxis (cohort A) or treatment (cohorts B and C) of CNS involvement. Mean patient age was 47 years (range 18–79), 60 % (*n* = 72) were males, and the most common histological subtype was DLBCL (diffuse large cell lymphoma, *n* = 80, 67 %). Overall, 89.2 % (*n* = 107) of cases were treated at diagnosis, while 10.8 % (*n* = 13) were treated for disease relapse. Patients’ baseline characteristics are summarized in Table 1. Patients in cohort A (*n* = 88) were diagnosed with lymphoblastic (LB) or Burkitt’s (BL) lymphomas (*n* = 13, 14.8 %) or high-risk DLBCL or other aggressive lymphomas (*n* = 75, 85.2 %). None of them had any symptoms or signs of CNS involvement, nor increased pleocytosis in cerebrospinal fluid (CSF) analysis. ‘High-risk’ DLBCL or other aggressive NHL was defined as: lymphoma cell infiltration at specific sites (vertebral column, orbits, sinuses, testes: *n* = 17, 19.3 %) or existence of at least 2 out of 3 recognized risk factors: an IPI score of 3–5; elevated serum LDH; involvement at ≥2 extranodal sites, (*n* = 68, 77.3 %). They received prophylaxis with intrathecal liposomal cytarabine, 50 mg every 2–4 weeks.

**Table 1:** Baseline demographics and characteristics

Baseline parameter	All patients (*n* = 120)	Prophylaxis: cohort A (*n* = 88)	Treatment: cohort B (*n* = 7)	Treatment: cohort C (*n* = 25)
Demographics				
Mean age (years)	48	47	48	50
Male [*n* (%)]	72 (60)	57 (65)	2 (29)	13 (52)
Female [*n* (%)]	48 (40)	31 (35)	5 (71)	12 (48)
Lymphoma type [*n* (%)]				
Diffuse large B cell lymphoma	80 (67.0)	62 (70.5)	6 (85.7)	12 (48.0)
Lymphoblastic lymphoma	11 (9.0)	10 (11.4)	0 (0.0)	1 (4.0)
Primary mediastinal B cell lymphoma	8 (7.0)	6 (6.8)	1 (14.3)	1 (4.0)
Primary CNS lymphoma	8 (7.0)	0 (0.0)	0 (0.0)	8 (32.0)
Peripheral T cell lymphoma	5 (4.0)	5 (5.7)	0 (0.0)	0 (0.0)
Burkitt’s lymphoma	4 (3.0)	3 (3.4)	0 (0.0)	1 (4.0)
Mantle cell lymphoma	4 (3.0)	2 (2.3)	0 (0.0)	2 (8.0)
Systemic + measurable CNS disease [*n* (%)]	14 (11.7)	0 (0.0)	0 (0.0)	14 (56.0)
Isolated measurable CNS disease, *n* (%)	11 (9.2)	0 (0.0)	0 (0.0)	11 (44.0)
High risk (2–3 risk factors^a^) [*n* (%)]	84 (70)	68 (77.3)	7 (100)	9 (36)
Two risk factors	57 (47.5)	51 (58.0)	2 (28.6)	4 (16.0)
Three risk factors	27 (22.5)	17 (19.3)	5 (71.4)	5 (20.0)
High risk: Special extranodal localization^b^	30 (25.0)	17 (19.3)	2 (28.6)	11 (44.0)
High risk: Diagnosis (lymphoblastic and Burkitt’s lymphoma)	15 (13.0)	13 (14.80)	0 (0.0)	2 (8.0)
Pleocytosis (lymphocytes > 15/μl) [*n* (%)]	25 (20.8)	0 (0.0)	7 (100 %)	21 (84 %)

*CSF* cerebrospinal fluid, *CNS* central nervous system

^a^Risk factors defined as International Prognostic Index score 3ne-belevated lactate dehydrogenase, and ≥2 extranodal sites

^b^Patients with involvement at special sites, including infiltration of vertebral column, orbits, sinuses, or testes were also considered at high risk

Patients in cohort B (*n* = 7) had measurable systemic disease and clinically important CSF pleocytosis, defined as a lymphoma cell count >15/µl (mean 39 cells/µl, range 15–164/µl) without neurological symptoms or radiological CNS involvement. They received intrathecal liposomal cytarabine, 50 mg every 2–4 weeks with curative intent, plus concomitant non-CNS-penetrating systemic chemotherapy.

Patients in cohort C (*n* = 25) had CNS involvement diagnosed on imaging studies (eight in CT, ten in NMR, seven in CT and NMR). Clinically important neurological signs and symptoms were present in 23 (92 %) of the cases. Most of the patients (*n* = 21, 84 %) in this group had an important pleocytosis (>15 cells/µl, mean 400/µl, range 21–4000/µl). Eleven patients (44 %) had primary and 14 (56 %) secondary CNS involvement.

Each cohort received intrathecal liposomal cytarabine, 50 mg every 2–4 weeks. The mean time from diagnosis to the first intrathecal liposomal cytarabine injection ranged from 78.5 days in prophylaxis cohort A to 177 days in cohort C. The mean number of intrathecal liposomal cytarabine injections was 3.1, 4.0, and 4.3 in cohorts A, B, C, respectively. Therapy administered in subsequent groups is summarized in Table 2.

**Table 2:** CNS disease type and treatment/prophylaxis details

Baseline parameter	All patients (*n* = 120)	Prophylaxis: cohort A (*n* = 88)	Treatment: cohort B (*n* = 7)	Treatment: cohort C (*n* = 25)
Proportion treated for de novo disease [*n* (%)]	107 (89.2)	84 (95.5)	6 (85.7)	17 (68.0)
Proportion treated for relapsed disease [*n* (%)]	13 (10.8)	4 (4.5)	1 (14.3)	8 (32.0)
Mean time from diagnosis to first liposomal cytarabine injection (days)	136	78.5	116	177
Number of liposomal cytarabine injections [mean (range)]	3.4 (1–8)	3.1 (1–7)	4.0 (1–7)	4.3 (2–8)
Systemic low-dose chemotherapy [*n* (%)]	94 (78.3)	76 (86.3)	7 (100.0)	11 (44.0)
R-CHOP	91 (75.8)	73 (83.0)	6 (85.7)	9 (36.0)
R-CVP	4 (3.3)	2 (2.3)	0 (0.0)	2 (8.0)
Other chemotherapy	3 (2.5)	1 (1.1)	1 (14.3)	0 (0.0)
Systemic, CNS-penetrating chemotherapy [*n* (%)]	25 (20.8)	12 (13.6)	0 (0.0)	13 (52.0)
R-CODOXM/R-IVAC (in BL)	3 (2.5)	2 (2.3)	0 (0.0)	1 (4.0)
EVAP (in LBL)	8 (6.7)	8 (9.1)	0 (0.0)	0 (0.0)
R-CHOP/RHAD (in MCL)	2 (1.7)	2 (2.3)	0 (0.0)	0 (0.0)
MA/IVAC	7 (5.8)	0 (0.0)	0 (0.0)	7 (28.0)
ESHAP	2 (1.7)	0 (0.0)	0 (0.0)	2 (8.0)
RHAD	1 (0.8)	0 (0.0)	0 (0.0)	1 (4.0)
R-CHOP/MTX	1 (0.8)	0 (0.0)	0 (0.0)	1 (4.0)
IVAC	1 (0.8)	0 (0.0)	0 (0.0)	1 (4.0)
Whole brain radiation therapy [*n* (%)]	18 (15.0)	0 (0.0)	0 (0.0)	18 (72.0)
Autologous stem cell transplant post-chemotherapy (consolidation or relapse)	19 (15.8)	16 (18.2)	0 (0.0)	3 (12.0)

*BL* Burkitt’s lymphoma, *CNS* central nervous system, *CT* chemotherapy, *ESHAP* etoposide combined with solumedrol, high-dose cytarabine and platinum-based chemotherapy, *EVAP* etoposide combined with vinblastine, adriamycin and prednisolone in patients with LBL, *IVAC* ifosfamide, etoposide and high-dose cytarabine, *LBL* lymphoblastic lymphoma, *MA/IVAC* methotrexate and cytarabine administered in alternate cycles with IVAC, *MCL* mantle cell lymphoma, *R-CHOP* rituximab combined with cyclophosphamide, hydroxydaunorubicin, vincristine (Oncovin) and prednisone, *R-CODOXM/R-IVAC* rituximab plus cyclophosphamide, vincristine, doxorubicin, and high-dose methotrexate followed by rituximab in combination with IVAC in patients with BL, *R-CVP* rituximab plus cyclophosphamide, vincristine and prednisone, *R-CHOP/RHAD* R-CHOP followed by rituximab plus high-dose cytarabine according to the Nordic protocol in patients with MCL

## Results

### Toxicity evaluation

The overall AE incidence was 79.2 %; however, the majority of AEs were mild-to-moderate in severity (grades 1–2); none of the patients developed grade 4 AEs (Table 3). The most common AEs were headaches (69.2 %), nausea (20.8 %), and fever (16.7 %). The incidence of headaches was higher in cohort B than cohorts A and C (85.7 vs. 64–69 %), while nausea (40 vs. 14.8–28.6 %) and neurological deficits (28 vs. 0–3.4 %) were more frequently reported in cohort C than cohorts A and B (Table 1). The incidences of fever (24–28.6 vs. 13.6 %) and dizziness (14.3–16 vs. 3.4 %) were higher in cohorts B and C than in cohort A (Table 3). Myelopathy (cauda equine syndrome) was reported in two cases (one in cohort A, one in cohort C, 1.1 and 4 %, respectively). In both cases, it was mild (CTCAE grade 1), described as saddle paraesthesia involving S4–S5 dermatomes, without incontinence and motor dysfunctions. It resolved without any dedicated treatment after 2 and 3 days. Transitory neurological deficits were described in 10 cases (three patients with CTCA grade 2, 7 with grade 3): three in cohort A and seven in cohort C. In total, four cases of arachnoiditis (grade 2 and 3) were reported after liposomal cytarabine, including two patients treated with systemic high-dose cytarabine and MTX in cohort C and two patients receiving CNS prophylaxis, while on R-CHOP in cohort A. Arachnoiditis was diagnosed in patients where important (grades 2–3) headaches were accompanied by fever, neck stiffness, nausea, and vomiting. One arachnoiditis was described after the first, two after the third, and one after the fourth Depocyte dose.

**Table 3:** Summary of adverse events reported in cohorts A–C

Adverse events	All patients (*n* = 120)	Prophylaxis: cohort A (*n* = 88)	Treatment: cohort B (*n* = 7)	Treatment: cohort C (*n* = 25)
*Individual adverse events (CTCAE any grade)*				
Adverse events (any grade) [*n* (%)]	95 (79.2)	69 (78.4)	6 (85.7)	20 (80.0)
Headache [*n* (%)]	83 (69.2)	61 (69.3)	6 (85.7)	16 (64.0)
Nausea [*n* (%)]	25 (20.8)	13 (14.8)	2 (28.6)	10 (40.0)
Fever [*n* (%)]	20 (16.7)	12 (13.6)	2 (28.6)	6 (24.0)
Neurological deficits [*n* (%)]	10 (8.3)	3 (3.4)	0 (0.0)	7 (28.0)
Dizziness [*n* (%)]	8 (6.7)	3 (3.4)	1 (14.3)	4 (16.0)
Vomiting [*n* (%)]	8 (6.7)	5 (5.7)	1 (14.3)	2 (8.0)
Myelopathy [*n* (%)]	2 (1.7)	1 (1.1)	0 (0.0)	1 (4.0)
*Individual adverse events (CTCAE grade 3)*				
Grade 3 events [*n* (%)]	7 (5.8)	6 (6.8)	0 (0.0)	1 (4.0)
Headaches [*n* (%)]	5 (4.1)	4 (4.5)	0 (0.0)	1 (4.0)
Fever	1 (0.8)	1 (1.1)	0 (0.0)	0 (0.0)
Nausea	1 (0.8)	1 (1.1)	0 (0.0)	0 (0.0)

Only seven patients developed grade 3 AEs: five headaches, one fever, one nausea: six of them were reported in cohort A (CNS prophylaxis), one in cohort C (CNS lymphoma therapy)

Only seven (5.8 %) patients developed grade 3 AEs: five (4.1 %) headaches, one (0.8 %) fever, one (0.8 %) nausea: six of them were reported in cohort A (CNS prophylaxis) and one in cohort C (CNS lymphoma therapy). There were no grade 3 AEs in cohort B, compared with 4.0 % in cohort C and 6.8 % in cohort A.

The frequency of mild-to-moderate (CTCAE grades 1–2) AEs did not increase with the number of Depocyte intrathecal injections; however, grade 3 events usually happened later, after the second (mean fourth) administration. The incidence of AEs was not correlated with the frequency of lumbar punctures: 81 versus 67 % (P–NS) for patients exposed to Depocyte every 2 and 4 weeks, respectively. AEs were seldom the cause of premature treatment/prophylaxis termination.

### Cytological response

None of the 88 patients to whom intrathecal cytarabine were given as prophylaxis, developed pleocytosis, nor did they incur CNS relapse at the average follow-up of 3 years. Although the CSF assessment was made using the cytological method, which is less specific than flow cytometry, the length of response provides the additional confirmation of Depocyte efficacy. Twelve patients (13.6 %) received induction concomitant systemic chemotherapy in doses penetrating to the CNS, and a further 16 (18.2 %) were treated with high-dose chemotherapy supported by autologous stem cell transplants for systemic relapse.

In cohort B (mean pleocytosis 39 cells/µl, range 15–164/µl), the cytological response was excellent, and our observations confirmed the efficacy of Depocyte. In 3/7 cases, a complete response was observed after the first, in 2/7 case after the second and in 2/7 cases after the third intrathecal administration. As responses were not assessed by flow cytometry, it was recommended to carry on with therapy and to administer four doses (range 2–6).

In cohort C, CSF involvement was documented in 21/25 (84 %) cases (mean pleocytosis 400/µl, range 21–4000/µl). Complete cytological remission was obtained in 16 cases (at the end of therapy 20/25 patients had a cytological CR). A summary of the cytological response rates is shown in Table 4.

**Table 4:** Summary of cytological response rates.^a^

Cytological (CSF) response	Treatment: cohort A (CSF pleocytosis, *n* = 7)	Treatment: cohort B (Measurable CNS involvement, *n* = 25)
Response (CSF count < 15 lymphoma cells/ml) [*n* (%)]	7 (100)	21 (84)
Failure (CSF count ≥ 15 lymphoma cells/ml) [*n* (%)]	0 (0)	5 (16)

*CNS* central nervous system, *CSF* cerebrospinal fluid

^a^CSF cell count normalization (response < 15 cells/μl) or clinically important pleocytosis (failure ≥ 15 cells/μl) in cohorts A and B

### Response assessment

**Fig. 1:**
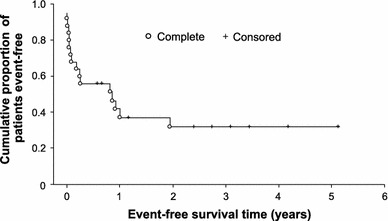
Event-free survival in patients in cohort C* (*n* = 25)

In total, 98 of 120 (82 %) patients were still alive after an average follow-up of 3 years, including 77 (87.5 %) in cohort A, all 7 (100 %) patients in cohort B and 14 (56 %) in cohort C:

- Cohort A: at three-year follow-up, PFS and OS were 80 and 88 %, respectively. More detailed efficacy analysis of CNS relapse prophylaxis in this group has been addressed previously, in a different paper [24].
- Cohort B: median OS and EFS had not been reached at three-years follow-up (all patients achieved CR, all were alive, none had progressed). With seven patients included in this cohort, no conclusions can be drawn; however, our observation confirmed the exceptional efficacy of liposomal cytarabine in this clinical setting (registration indication).
- Cohort C: Clinical responses were observed in 19 of 25 (76 %) patients, including 10 (40 %) complete responses (CR) and 9 (36 %) partial responses (PR). Neurological response rates (72.7 and 78.6 %) were similar in lymphoma patients with primary and secondary CNS involvement. The response rate was 75 % in patients with DLBCL (41.7 % CR) and PCNSL (50 % CR); other histological subtypes were too rare to assess them separately (Table 5). The response rate was 66.6 % (16.6 % CR and 50.0 % PR) with CNS-directed therapy with liposomal cytarabine + WBRT (±low-dose chemotherapy). A similarly high neurological response rate was achieved with liposomal cytarabine plus high-dose CNS-penetrating chemotherapy with (85.7 %, including 57.1 % CR) or without (83.3 %, including 66.6 % CR) WBRT. Median EFS was 10 months (Fig. 1), and median OS was 4.8 years.

**Table 5:** Summary of clinical responses by lymphoma type or concomitant therapy

Cohort B: overall responses	ORR *n* (%)	CR *n* (%)	PR *n* (%)	SD *n* (%)	PD *n* (%)
All patients (*n* = 25)	19 (76.0)	10 (40.0)	9 (36.0)	2 (8.0)	4 (16.0)
Responses by individual lymphoma type					
Diffuse B cell lymphoma (*n* = 12)	9 (75.0)	5 (41.7)	4 (33.3)	1 (8.3)	2 (16.7)
Primary CNS lymphoma (*n* = 8)	6 (75.0)	4 (50.0)	2 (25.0)	1 (12.5)	1 (12.5)
Mantle cell lymphoma (*n* = 2)	2 (100.0)	0 (0.0)	2 (100.0)	0 (0.0)	0 (0.0)
Burkitt’s lymphoma (*n* = 1)	1 (100.0)	1 (100.00)	0 (0.0)	0 (0.0)	0 (0.0)
Lymphoblastic lymphoma (*n* = 1)	1 (100.0)	0 (0.0)	1 (100.0)	0 (0.0)	0 (0.0)
Primary mediastinal B cell lymphoma (*n* = 1)	0 (0.0)	0 (0.0)	0 (0.0)	0 (0.0)	1 (100.0)
Responses by regimen added concomitantly with liposomal cytarabine					
WBRT (±low-dose systemic chemotherapy) (*n* = 12)	8 (66.6)	2 (16.6)	6 (50.0)	2 (16.6)	2 (16.6)
High-dose CNS-penetrating chemotherapy (*n* = 6)	5 (83.3)	4 (66.6)	1 (16.7)	0 (0.0)	1 (16.7)
WBRT + high-dose CNS-penetrating chemotherapy (*n* = 7)	6 (85.7)	4 (57.1)	2 (28.6)	0 (0.0)	1 (14.3)

*CNS* central nervous system, *CR* complete response, *ORR* objective response rate, *PR* partial response, *PD* progressive disease, *SD* stable disease, *WBRT* whole brain radiation therapy

## Discussion

Liposomal cytarabine has favourable pharmacokinetics and possibly better biodistribution in comparison with intrathecal MTX and cytarabine. Due to liposomal formulation, its half-life in CSF is more than 30 times longer (141 h compared to 3.4 h and 4.5 h for MTX and cytarabine, respectively). In this paper, we have addressed the feasibility and toxicity issues. The retrospective nature of our study was not designed to make definite clinical recommendations.

The relatively short time interval from initial diagnosis of NHL to CNS involvement suggests that seeding of the CSF with malignant lymphoma cells occurs early in the natural course of the disease [25, 26]. Therefore, prophylaxis or ‘early treatment’ could help prevent/reduce leptomeningeal and/or parenchymal CNS involvement in patients with systemic disease. The necessity of prophylaxis—indisputable in lymphoblastic and Burkitt lymphomas—depends on proper identification of the high risk group in other lymphoma subtypes. There seems to be no difference of the incidence of CNS involvement between DLBCL, primary mediastinal B cell lymphoma or peripheral T cell lymphomas (including anaplastic T large cell, angioimmunoblastic T cell lymphoma, and others) [27]. In our study, those subjected to CNS prophylaxis only included patients with well-established risk factors: “specific localizations” or at least two of the following three features: IPI 3–5, elevated LDH, 2 or more of extranodal localizations [28]. As “specific localizations”, we regarded only lymphoma infiltration of the testis, breast, epidural space, or cranial air sinuses [29–32]. Such an approach was consistent with guidelines published recently in BJH, with a level of evidence of 1b: one could have expected 7–15 % incidence of CNS relapse in those patients [33].

A recent retrospective database analysis evaluated CNS relapse rates in 435 high-risk aggressive lymphoma patients who mostly (>95 %) received systemic chemotherapy without prophylaxis [16]. Over two-thirds of CNS relapses occurred within the first year of diagnosis, the overall CNS relapse rate was 7.1 %, and the median time to CNS relapse was 8.1 months with CHOP and 6.7 months with R-CHOP treatment. The projected three-year CNS relapse rates were 6.4 % with R-CHOP and 9.7 % for CHOP, respectively [16].

Our data which show no relapses after 3 years confirm the efficacy of intrathecal liposomal cytarabine prophylaxis, but due to the retrospective nature of this study, cannot be used to make definite clinical recommendations. However, it should be noted that currently, we do not have a widely accepted standard, and the level of evidence of the most commonly used 3–6 cycles of intrathecal MTX is only 2c.

The major strength of this paper is the toxicity analysis—with 120 patients, including 88 with intrathecal liposomal cytarabine used in prophylactic setting, it is one of the largest series published so far. In Polish Lymphoma Research Group retrospective analysis liposomal cytarabine was well-tolerated; although 79.2 % of patients had at least one AE, the vast majority were grades 1–2 in severity. The most common AEs were headaches (69.2 %), nausea (20.8 %), and fever (16.7 %), and the incidence of transitory neurological deficits (8.3 %), dizziness (6.7 %) and myelosuppression (1.7 %) was low. There were four cases of arachnoiditis, which has been raised as a concern with liposomal cytarabine; however, none were grade 4 or higher in severity (*n* = 2 grade 3; *n* = 2 grade 2). They all occurred after the third dose of the drug. Arachnoiditis can be prevented by oral or intravenous steroids or by reducing the dose of liposomal cytarabine to 25 mg and, perhaps, by reducing the number of doses applied. The incidence of cauda equine syndrome was low in our series: one case in cohort A (1.1 %) and one in cohort C (4 %). They were both mild and transitory, but there are AEs described in the literature as potentially irreversible. It has been shown in a retrospective case series that 23.3 % of patients experienced Common Toxicity Criteria ≥ Grade 3 neurotoxicity related to the Depocyte injections. 43 % of these AEs were permanent and may have impacted patients’ quality of life [34]. In another small retrospective analysis, four of fourteen patients (28 %) with high-grade NHL that received prophylactic therapy with IT liposomal cytarabine developed moderate or severe neurotoxicity (grades 2 and 3 of the National Cancer Institute Common Toxicity Criteria), manifested as conus medullaris/cauda pseudotumour cerebri-like syndrome, after a median of 3.5 intrathecal courses of liposomal cytarabine [35]. One case of fulminant chemical ventriculomeningitis following intrathecal liposomal cytarabine administration has been described in the literature [36]. The aforementioned AEs are rare, but to avoid them, the careful choice of which patients to subject to liposomal cytarabine for CNS prophylaxis is necessary.

It is important to mention that all adverse events (CTCAE 1–3) were evenly distributed in each of the cohorts (78.4, 85.7, and 80 % in cohorts A, B, and C subsequently). It is also true for important, grade 3 events, which occurred in 6.8 % (6/88) patients prophylaxed with Depocyte. Although they all resolved with time and symptomatic treatment, they may be potentially dangerous, causing long-lasting neurological consequences. It is a debatable whether such a possibility, which is fully acceptable for patients treated for CNS involvement (cohorts B and C), is allowed for prophylactic settings.

No CTCAE grade 4 AEs and the relatively low incidence of grade 3 adverse reactions in our series could have been due to: mandatory hospitalization allowing for proper hydration prior to lumbar puncture, recommended horizontal position for 6–8 h after procedure (also important for improving intraventricular drug perfusion) [37] and concomitant chemotherapy regimens with systemic steroids. To improve lumbar puncture feasibility atraumatic, noncutting spinal needles (22 gauge or lower) should be used. They decrease the risk of CSF leaks and postlumbar puncture headaches [38] and may also prevent cerebral bleeding or thrombosis [39]. The early removal of the stylet (after passage through the epidermal and subcutaneous tissues) may significantly improve the success rate of lumbar punctures [40].

An important question which needs to be addressed is the optimal intrathecal dosing schedule. It should be mentioned, that the optimal Depocyte dosing schedule is not yet established: neither the optimal dose (50 mg may not be necessary), nor its frequency (every 4 weeks seems in our data as effective as administered with 2-week intervals) or eventually the number of intrathecal administrations is known. The number of Depocyte doses may correlate with the incidence of arachnoiditis. In our study, we aimed to administer four doses at the time of systemic therapy. Although the regimen was well-tolerated, 13 (15 %) patients received three and 29 (33 %) received only two doses in our study. The main reason why patients did not complete the planned number of intrathecal injections was the inconvenience of having to stay in hospital overnight after the lumbar puncture as well as the physician’s decision. Interestingly, high cytological and neurological response rates were still achieved despite the fact that many patients received a lower than planned number of liposomal cytarabine injections.

The cytological response was restrictive. Although it was assessed using the cytological method which was repeated at each intrathecal administration, and not by more specific flow cytometry, the length of CNS progression-free survival in cohorts A and B is an additional argument for Depocyte efficacy. None of those patients progressed. In Cohort C, the cytological response rate of 84 % is above expectation.

NHL patients with CNS involvement (cohort C) who typically have a very poor prognosis also appeared to benefit from additional intrathecal liposomal cytarabine, with a 76 % neurological response rate, 40 % CR rate, and a striking 4.8-year median OS. Notably, although sample numbers were small, the highest neurological overall response rate (ORR) and CR rates were achieved in patients who received intrathecal liposomal cytarabine and high-dose CNS-penetrating chemotherapy. No additional benefit was apparent when WBRT was added to this regimen. The toxicity of regimens containing liposomal cytarabine is acceptable; thus, the intrathecal liposomal cytarabine plus high-dose chemotherapy regimen may avoid the toxicities of WBRT without compromising efficacy.

## Conclusions

Our analysis has demonstrated the three-yearyear efficacy of liposomal cytarabine regimens in the prophylaxis or treatment of CNS involvement in high-risk patients with aggressive NHL. The toxicity profile was acceptable, even in a prophylaxis setting; thus, allowing the recommendation of liposomal cytarabine in chosen, high-risk patients.
